# Extended Interviews with Stroke Patients Over a Long-Term Rehabilitation Using Human–Robot or Human–Computer Interactions

**DOI:** 10.1007/s12369-022-00909-7

**Published:** 2022-09-16

**Authors:** Yaacov Koren, Ronit Feingold Polak, Shelly Levy-Tzedek

**Affiliations:** 1 Department of Sociology and Anthropology, Tel-Aviv University, Tel-Aviv, Israel; 2 Recanati School for Community Health Professions, Department of Physical Therapy, Ben-Gurion University of the Negev, Beer-Sheva, Israel; 3 Zlotowski Center for Neuroscience, Ben-Gurion University of the Negev, Beer-Sheva, Israel; 4 Freiburg Institute for Advanced Studies (FRIAS), University of Freiburg, Freiburg, Germany

**Keywords:** Socially assistive robot (SAR), Stroke rehabilitation, Long-term interaction, Trust, Qualitative methods, In the wild

## Abstract

Socially assistive robots (SARs) have been proposed to assist post-stroke patients in performing their exercise during their rehabilitation process, with the trust in the robot identified as an important factor in human–robot interaction. In the current study, we aimed to identify and characterize factors that influence post-stroke patients’ trust in a robot-operated and a computer-operated rehabilitation platform during and after a long-term experience with the platform. We conducted 29 interviews with 16 stroke patients who underwent a long-term rehabilitation process, assisted by either a SAR or a computer interface. The intervention lasted 5–7 weeks per patient, for a total of 229 sessions over 18 months. By using a qualitative research method—extended interviews “in the wild” with stroke patients, over a long-term rehabilitation process—our study reveals users’ perspectives regarding factors affecting trust in the SAR or in the computer interface during their rehabilitation process. The results support the assertion that SARs have an added value in the rehabilitative care of stroke patients; It appears that personal characteristics, such as age and gender, have an effect on the users’ acceptance of a non-human operator as a practice assistant. Our findings support the notion that SARs augment rehabilitative therapies beyond a standard computer; Importantly, patients appreciated different aspects of the non-human operator in the two groups: In the SAR group, users preferred its functional performance over its anthropomorphized social skills; In the Computer group, users highlighted its contribution to the training of their memory skills.

**Supplementary Information:**

The online version contains supplementary material available at 10.1007/s12369-022-00909-7.

## Introduction

The use of robots in healthcare represents an opportunity to support a large number of people, some of whom may suffer from cognitive, sensory, and motor impairments [1]. Socially assistive robots (SARs) have been proposed as a tool to help individuals who have had a stroke to perform their exercise during their rehabilitation process [2] to augment the current range of therapeutic options [3]. It has been suggested that SARs can be incorporated into the rehabilitation practice regime that calls for repetitive tasks in order to increase stroke patients’ motivation [4–7]. Trust in robots, namely the level of how much users rely on the system to achieve its goal [8], is a key factor of human–robot interaction (HRI) relationship [8, 9]. This is especially important in healthcare scenarios [10, 11] involving vulnerable populations, such as neurologically impaired patients, where establishing a long-term trust between the patient and the robot is essential for its acceptance, its use, and for maintaining an ongoing rehabilitative training regime [3, 12]. Langer and colleagues [3] outlined a framework of guidelines and considerations when designing a socially assistive robot (SAR) for use in rehabilitation, and highlighted factors which most likely influence trust in SARs in this unique context. In the current study, we investigate the factors that affect HRI trust when a user (a person with stroke) is in direct interaction with a non-human operator—either a SAR (the humanoid robot Pepper) or a computer interface [2, 13]—as part of a gamified system for post-stroke rehabilitation (the “system” or “platform”). The goal of this investigation is to identify the factors that contribute to the formation of trust when a SAR, or a computer interface, are used in rehabilitation. We also aim to add a complementary empirical layer of knowledge, based on a user-centered approach [3, 12, 14], about users’ preferences, while and after undergoing a long-term rehabilitation process with a non-human operator. Our specific objectives in the current analysis were to identify and characterize: (1) the users’ perspectives and evaluation of the SAR/computer-based system in a long-term rehabilitation process (15 exercise sessions conducted over 5–7 weeks); (2) users’ relative perception of HRI vs. human–computer interaction (HCI) in post-stroke rehabilitation; (3) factors that influence post-stroke patients’ trust in an HRI/HCI-based rehabilitation system, during and after a long-term intervention experience with the system; (4) the users’ terms of acceptance, and grounds for rejection of this technology.

To that end, we conducted a series of 29 interviews in order to gather patients’ unstructured and detailed feedback and impressions. We interviewed users from two groups of patients: one group received instructions and feedback during the 15 rehabilitation-exercise sessions from a humanoid robot, and the other used the exact same exercise platform, but received instructions and feedback from a computer. Our goal in interviewing both groups was to explore differences between them in attitudes to the non-human operator. The study introduces an in-depth qualitative study [15] using extended interviews with stroke patients during and following an “in the wild” intervention; that is, an intervention that takes place in the natural setting in which these interactions will eventually occur [16, 17]. It contributes an additional methodological tool and empirical knowledge to the research of non-human operator platforms (SAR/computer), to be used by developers of rehabilitation platforms for stroke patients.

## Previous Related Work

### Trust in HRI

Users’ trust, and factors affecting it, have been explored in relation to various forms of technologies, such as trust in automation [18–20], artificial intelligence (AI) [21], decision support systems (DSS) [22], and robots [9, 23], to name a few. Yet, each type of technology is distinct [21].

The notion of “trust” in the context of HRI is ambiguous and not unequivocally defined [3, 23–26]. There are several competing definitions of trust, and a consensus has not been reached on one specific definition of the concept [21, 27]. A common general definition used by HRI roboticists, regardless of the specific operational context [9], delineate HRI trust as the willingness of people to accept robot suggestions and follow their instructions [3, 23]. Lee and See [28] defined trust as the attitude that an agent will help achieve a person’s goals in a situation characterized by uncertainty and vulnerability. Hancock and colleagues [23] define trust as “the reliance by an agent that actions prejudicial to their well-being will not be undertaken by influential others”. According to Kim et al. [8], “Trust in HRI is defined as a measure of how much users rely on an automated system to achieve a goal and is one of the most important metrics for evaluating a robotic system”. In contrast, Chiou and Lee [18] criticize “reliance” as a trust factor, favoring a “relational approach”. They claim that in complex work environments, automation has become more autonomous in recent years. Robots may be working with people more as coworkers than as tools. In such situations, automation responsivity and the ability to resolve conflicting goals may be more relevant than reliability and reliance. Chiou and Lee [18] agree that the traditional concept of trust [23] should be applied in well-defined (narrowly scoped) human-automation tasks. As HRI in rehabilitation is a unique, well-defined context, we used the traditional concept of trust in this work. Furthermore, the population we studied (post-stroke patients) is characterized by uncertainty and vulnerability, which further supports the use of the approach outlined by Lee and See [28]. Thus, for the purpose of this study, we defined trust in HRI as the level of how much users rely on the system to achieve its goal [8, 9].

### Factors Affecting Trust in HRI

Researchers constantly examine prospective factors that affect trust in robots, generally categorized to: robot-related, user-related, or pertaining to contextual environment [3, 9, 20, 23, 25]. In a meta-analysis of studies on trust in HRI, Hancock et al. [23] found that robot performance-related factors (e.g., reliability, failure rate) had the greatest influence on developing trust in the robot. The study of Natarajan and Gombolay [29] revealed the importance of the robot’s behavior and anthropomorphism as a significant factor in predicting the trust in the robot and compliance with it. Initially, factors related to the user (e.g., prior experience, personality traits, propensity to trust) were not found to be significantly associated with trust [23]. However, later, Schaefer et al. [20] found that user-related factors affect trust development in human–automation interaction. In a recent meta-analysis, Hancock et al. [9] confirmed their previous claim that factors relating to the robot have more impact on trust than factors relating to the human. However, in addition to the robot factors and the context, they identified that human-related factors, like satisfaction with a robot interaction, are significant factors affecting HRI trust as well.

### Factors Affecting Trust in SAR in a Rehabilitation Context

One of the applications of SAR is in the field of medical rehabilitation is to address the care gaps in the rehabilitation of patients with severe functional impairments, due to, for example, a stroke [12]. Though SAR systems are not expected to replace therapists in the rehabilitation context [2, 4, 12, 13], they are intended to interact with patients in the absence of a therapist, for instance to encourage practice in between individual sessions with a therapist [4]. Thus, the SARs used in rehabilitation must be trusted not only as therapeutic tools, but also as social entities. Therefore, it was argued that both the robot’s technical ability and its social behavior contribute to the trust relationship formed between patients and the SAR in rehabilitation [3]. In order for a SAR to be trusted as a partner during rehabilitation, it should be able to produce human-like behavioral cues [30], such as gestures and facial expressions, and exhibit social signals [3].

Hancock et al. [9, 23] provide a comprehensive meta-analysis on the antecedents of trust in HRI. Langer et al. [3] followed Hancock et al. [23] and related literature (e.g., [30–35]). They adapted the identified HRI trust factors to an HRI trust model applicable to “the context in which the automation is used” [28], namely to post-stroke rehabilitation objectives. They outlined a framework of guidelines and considerations when designing a socially assistive robot (SAR) for use in rehabilitation. Accordingly, they argue that in the context of rehabilitation, the main factors generating trust are: (1) the user’s confidence that the SAR can carry out its designated objective; (2) the rehabilitation robot’s “social character”; i.e., its human-like behavior and ability to produce recognizable intentions via gestures, facial expressions, and other human social skills; (3) the severity of a robot’s errors; (4) the SAR’s perceived safety; i.e., patients’ concerns regarding the safety of the robot; (5) the user’s prior experience with technology; (6) the user’s general propensity to trust people in general, and (7) the user’s “culture”—their national or ethnic background and worldview. Our study empirically and critically evaluates Langer et al.’s [3] analytical model from the users’ perspective.

Although previous studies [3–7, 12, 13] identified various factors affecting trust in SAR in a rehabilitation context, further empirical studies are required to expand knowledge and fill-in gaps, specifically—patients’ perspectives and perceptions following multiple interactions with the SAR in the wild. In addition, further empirical research is required in order to compare advanced rehabilitative care using a SAR versus other potential assistive technologies like a standard computer, in a Human–Computer-Interaction (HCI), in order to characterize whether SARs add value, and to what extent, like reported by Mann et al. [36] with regard to the provision of medical care instructions.

## Purpose of the Study

**Fig. 1:**
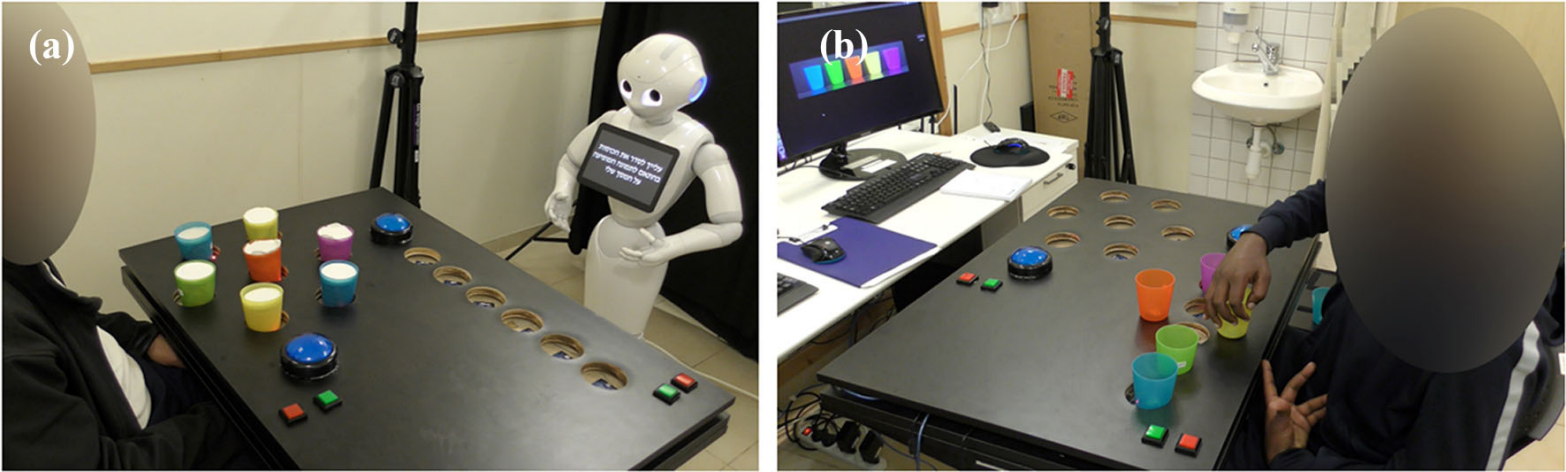
The experimental setup for the long-term post-stroke intervention. **a** Patient in the SAR group. **b** Patient in the Computer group

This study is part of a broader research project, a Randomized Control Trial (RCT) study, in which 33 participants were randomly allocated to three groups: two intervention groups and one control group. The experimental set-up with the intervention groups is shown in Fig. 1.

Participants in the intervention groups participated in 15 intervention sessions, three times a week over a period of 5–7 weeks. Each session lasted between 45 and 60 min. Clinical measures were taken before and at the end of the intervention and are not reported here. Participation in the study was in addition to the conventional rehabilitation program of the post-stroke patients in the ambulatory rehabilitation center Adi-Negev, which included physiotherapy, occupational therapy, hydrotherapy etc. The intervention program consisted of a set of gamified functional exercises for post-stroke upper-limb rehabilitation, where the instructions and feedback to users were provided by either a SAR (the robot “Pepper”, Softbank Robotics Aldebaran) or by a standard computer (for details on the system, see [13]).

The current study aims to empirically study users’ perspectives regarding factors affecting trust in SAR and in a computer operator in the context of post-stroke rehabilitation. In accordance with this goal, we interviewed individuals from the two intervention groups, and not individuals who were in the control group of the RCT study. Following the suggestion of Cameron et al. [24], for a bottom-up approach that emphasizes the importance of the user perspective in understanding trust in HRI, we present and analyze users’ viewpoint extracted from interviews, based on their real-life experience with the system in the wild [16, 17], and their attitude to this new innovative technological tool.

## Methods

The current study is based on qualitative methodology which supplements the RCT study [13]. Sixteen post-stroke patients who participated in the RCT study were also interviewed for this study. Unlike the RCT study, this study employs an in-depth qualitative analysis [15] using extended interviews as a single research method.

### Participants

A total of 16 post-stroke individuals from the participants who underwent intervention in the RCT study were interviewed (nine men, seven women; age range 30–74, mean 58 ± 13 years). Nine of those practiced with the system operated by SAR (five women, age range 30–68, mean 52 ± 11 years) and seven with the system operated by a computer (two women, age range 42–74, mean 66 ± 10 years). The inclusion criteria are detailed in the supplementary materials.

The study protocol was approved by the institutional Helsinki ethical committee for clinical trials (SMC-5273-2018). All participants gave their written informed consent after they received a detailed explanation of the study.

The research project involved post-stroke individuals with varying degrees of severity (e.g., mild, moderate, or moderate-severe impairment), which may have affected their comprehension and communication abilities. Six of the patients interviewed for this study suffered from mild speech or cognitive impairments, but with sufficient comprehension and communication competences. The special characteristics of the study population required methodological flexibility and modification to account for the needs of these vulnerable populations [16]. The patients were concentrated on their rehabilitation process. They were not always available, physically, cognitively, and emotionally to participate in the research, which required the investment of additional time and effort beyond the therapy process. However, the importance of running the current study with them, though operationally challenging, is that they reflect the target population of the system and context of usage. The study implements the “user-centered” approach in SAR design which considers and integrates the perspectives of the user’s personal and contextual factors [3, 12, 14].

### Procedure

This study is based on interaction–centered interviews (HRI and HCI) with post-stroke patients who participated in the RCT study and were asked to freely discuss their experience with the system, where the instructions and feedback were given by either a SAR or a computer. Interviews were carried out with 16 patients out of 20 who participated in either one of the intervention groups (SAR or Computer) of the RCT study. Other patients were not available for an interview due to medical conditions or other personal issues.

Qualitative research methods have been underutilized in HRI research [37]. This study is based on a qualitative method, interviews as a single research method. While interviews are not a common evaluation method in social robots’ usability studies, they are an appropriate way to evaluate trust in robots; this approach requires a “deep dive into users” [38]. We consider the implementation of a qualitative methodology, using multiple, in-depth interviews in the wild [16, 17], as a significant contribution to HRI research.

Interviews are an important source of insights and may provide perspectives and useful data that would otherwise be hard to capture. The conversation, and the questions and answers dialogue encourage reflection and consideration by interviewees, sharing insights that would have been lost in surveys [15]. The researcher can ask follow-up questions in response to users' answers, to better understand their intent [38]. In this context, the interview plan did not assume any predefined hypotheses regarding the HRI and the HCI (although it corresponded methodologically with previous literature and applied customary concepts) in order to deliver to the HRI community interviewees’ authentic opinions and insights.

Specifically, the interviews we conducted were aimed at identifying factors affecting trust in social robots in the specific context of post-stroke rehabilitation. Trust is especially important in healthcare scenarios [10, 11] involving vulnerable populations, such as neurologically impaired patients, where establishing a long-term trust between the patient and the robot is essential for its acceptance, its use, and for maintaining an ongoing rehabilitative training regime [3, 12]. Interviews are a tool to understand users’ viewpoints [15]. In this case to capture whether and to what extent acceptable HRI trust factors are indeed significant to users in the rehabilitation context, and to reveal unknown gaps in HRI trust literature in the rehabilitation context, based on patients’ real-life experience with the system in the wild [16, 17], and their attitude to this new innovative technological tool.

The semi-structured interview method we employed allows for open coding, emergent codes which are inductively derived from the transcripts, describing patients’ impressions of their experience with the system and their interpretations of that experience, which were not depicted by the a priori codes based on prior theory [15].

The relatively considerable number of interviews we conducted, above what is generally reported in a single study (see also Sect. 4.1), addresses the validation requirements for “multiple data sources”. This requirement can be met, as in this study, by “different instances of the same type of data [as] multiple participants in interview research” [15].

We employed “semi-structured interviews” [15] which include also open-ended questions. Those can be answered in a different way by each interviewee, which, on the one hand, enables the expression of a variety of views, but on the other hand, could be challenging when attempting to identify common grounds across participants [15]. In addition, in a semi-structured interview format, the researcher can collect information both on pre-defined issues of interest, as well as on issues that are important to the interviewee, which the researcher may not have considered.

The interviewer was not involved in the development of the system which helps to minimize bias [39]. Yet, it is important to emphasize that in general, qualitative analysis has a high subjective component, influenced by the researcher’s personal perspectives and biases, that may undermine the validity of the findings. However, it is not an impediment in this case, as the goal of the study is not to make a general claim, but rather, to understand users’ viewpoints [15] on factors affecting trust in SAR in the context of post-stroke rehabilitation, in order to present authentic evidence and insights that might facilitate understanding of other such cases as well [15].

### Data Collection

**Fig. 2:**
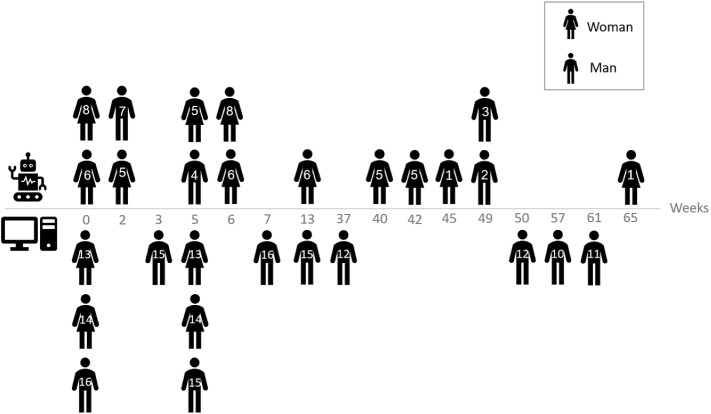
The distribution of interview times across the two groups. The horizontal axis denotes the weeks—since the start of the intervention—on which interviews were conducted. Above the axis are participants in the SAR group, and below it, are participants in the computer group. The numbers indicated on the silhouettes correspond to the participant number. The chart does not detail the interviews with a patient suffering from a severe speech dysarthria which was carried out by correspondence and lasted intermittently over approximately 2 months

The interviews were conducted throughout the period December 2019–October 2020. The location and duration of the interviews were adjusted to the constraints of the interviewees. Eight interviews were carried out face-to-face at the RCT research site (Adi Negev Rehabilitation Center), twenty by telephone (due to COVID-19 restrictions of social distancing) and one, with a patient suffering from a severe speech dysarthria, by correspondence. It should be noted that the imposed restriction of conducting the interviews remotely (due to COVID-19 restrictions) turned out to be beneficial, in that participants were able to schedule the interviews at a time that was convenient for them, rather than following an intervention session, after which they were often fatigued. All interviews were conducted in Hebrew, interviewees’ native language. In some cases, an interview was split into several sessions due to interviewee's constraints (for the distribution of interview times see Fig. 2).

The interviews lasted on average 47 ± 32 min and were audio recorded with interviewees’ prior consent. The interviews were transcribed verbatim by the interviewer. The semi-structured interview discussion included a set of pre-determined questions based on previous assumptions on factors influencing trust in SAR for post-stroke patient rehabilitation highlighted by Langer et al. [3]. The pre-determined questions, presented to patients in a language tailored to the interviewees’ capabilities [40, 41], were the following: (1) *Was the treatment with the SAR/computer helpful*? This question was designed to examine the assumed trust factor “functionality”; i.e., the patient’s faith in the SAR to perform its specific task; (2) *How was the social interaction with the SAR/computer*? This question was designed to examine the assumed trust factor “SAR’s social character”; Namely, the patient’s impression with its human-like behavior and ability to produce recognizable intentions via gestures, facial expressions, and other human social skills; (3) *Do you have previous experience with new technology?* (*e.g., the use of a computer or a smartphone*). This question was designed to examine the prevalence of the assumed trust factor “user’s prior experience with technology”; (4) *Were the failures of the SAR/computer disturbing*? This question was designed to examine the effect of the assumed trust factor “severity of the SAR’s errors”; (5) *Are you usually inclined to trust people*? This question was designed to examine the impact of the assumed trust factor “user’s general propensity to trust”; (6) *During the SAR/computer treatment, did you feel unsafe*? This question was designed to examine the effect of the assumed trust factor “SAR’s perceived safety”.

In addition, we asked open-ended questions [15] such as “what do you think of your exercise sessions with the SAR/computer?” to retrieve interviewees’ authentic opinions without imposing any external constraints on the responses. Questions were tailored to the interviewee, and unfolded as the conversation evolved, as is customary in semi-structured interviews [15]. Thus, questions sometimes varied from one interviewee to another. During each interview, individualized clarifying questions were used to better understand patients’ perspectives. An effort was made to allow a natural course of conversation to enable each interviewee to fully express herself, according to her verbal abilities, and alongside to advance the discovery of new insights. The common goal of all questions (pre-determined and open-ended) was to characterize post-stroke patients’ opinions on the factors which most likely influence trust when using this new rehabilitative technology in the long term.

### Data Analysis

#### Data Coding

Interview transcripts were analyzed to find recurring themes by coding the interviewees’ statements. The process of coding assigns labels to utterances from qualitative data forms. In this case by way of content analysis of interview transcripts which systematically compresses blocks of text [15] into fewer content categories based on explicit rules of coding [15, 42].

There are two different main approaches to analyzing the qualitative data: “a priori coding” and “emergent coding” [15]. A priori coding is based on an existing theoretical framework to guide the selection of coding categories. These categories might come from previously applicable published works. In contrast, emergent coding (open coding) refers to qualitative analyses conducted without any theory or model that might guide the analysis; The researcher scans the text systematically, looking for key items, making comparisons of data and nothing related concepts or ideas. While a priori coding parses the data according to existing theories, and either corresponds with them, confirms or contradicts them, the emergent coding approach enables the discovery of new insights.

#### Coding Procedures

The transcripts were analyzed using ATLAS.ti. The coding format we employed was a mixture of a priori and emergent coding, executed simultaneously. The a priori codes represent the structured component of the (semi-structured) interview and the emerging codes, its unstructured component. The a priori codes (robot-related and user-related factors), are based on factors marked in previous works as affecting trust in socially assistive robots (for a review, see [3]). The emergent codes (open coding) were inductively derived from the transcripts, describing patients’ impressions of their experience with the system and their interpretations of that experience, which were not depicted by the a priori codes. While a priori codes are a closed list (34 codes in this case), emerging codes is an open-ended list that deductively evolves, compared and modified in an iterative process at multiple levels throughout the coding process, which yielded, in this case, a total of 74 new codes.

The a priori codes are based on factors marked in previous works affecting trust in socially assistive robots in a rehabilitation context as detailed above. The a priori coding represent the structured component of the (semi-structured) interviews. They are the result of the answers to questions 1–6 detailed above. A priori coding parses the data according to existing theories and confirms or contradicts them. For each question and platform (SAR or Computer), three potential participant positions were identified in their answers and coded respectively as positive (supportive), negative (opposing), or neutral (ambivalent), for a total of six codes per question. So, for example, for the first question, “Was the treatment with the SAR/computer helpful?” for each of the SAR and Computer three codes were applied: positive (supportive)/negative (opposing)/neutral (ambivalent) trust in the rehabilitation system’s functionality. The reason that the total number of codes sums up to 34 and not 36 (six codes × six questions) is that for question number 6) (“SAR’s perceived safety”) only a subset of the codes was identified in participants’ responses.

**Fig. 3:**
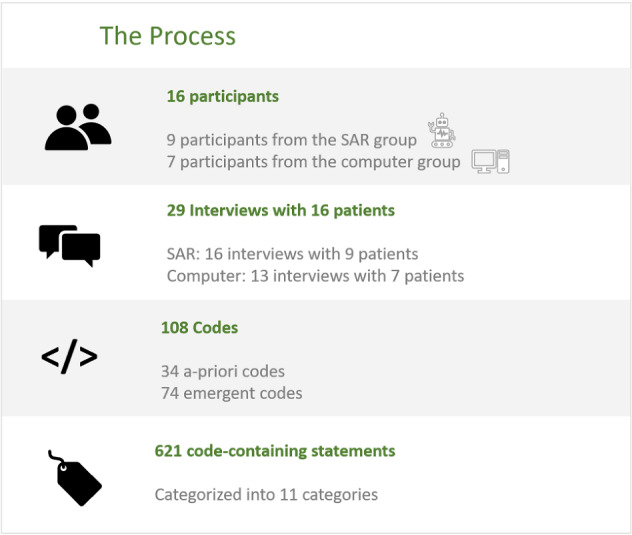
The interview and coding process

The coding process (see Fig. 3) yielded a total of 423 related statements from all 29 interviews. Codes, and their related statements, were grouped and classified into 11 main categories, each of which articulates a major theme identified in the interviews. Certain statements were applicable to more than one category and were classified accordingly, yielding finally 621 statements (see Table 1). Each of the 11 categories was divided into three sub-categories: supportive, opposing and ambivalent statements that express the patients’ attitude towards the discussed subject (see Table 2).

**Table 1:** Main coding categories

A priori coding			Open coding		
Category	Statements		Statements		
	#^a^	%^b^	Category	#^a^	%^b^
Trust in the rehabilitation system’s functionality	114	18	Patients’ satisfaction with the rehabilitation activity	118	19
Trust in the rehabilitation system’s social skills	81	13	Contribution to patients’ (hand) motor functions	54	9
Previous experience with technology	43	7	Contribution to patients’ cognitive (memory and spatial perception) functions	23	4
Tolerance to failures	33	5	Comparison to human therapist	93	15
Patients’ propensity to trust	8	1	Patient optimism, patience/pessimism, frustration	53	9
SAR’s perceived safety	1	–			
Total	280	44	Total	341	56

^a^Total number of statements coded for the category

^b^The percentage of statements, out of the total 621 coded statements, which contained a particular code – either based on a-priori coding (left), or on open coding (right)

**Table 2:** Main coding categories by patients’ opinion

Platform	SAR				Computer			
Category	Supporting	Opposing	Ambivalent	n/a	Supporting	Opposing	Ambivalent	n/a
Patients’ satisfaction with the rehabilitation activity	78%	22%	–	–	43%	14%	43%	–
	#45	#38	–	–	#14	#14	#7	–
Trust in the rehabilitation system functional skills	78%	22%	–	–	29%	29%	29%	14%
	#48	#26	–	–	#12	#18	#10	–
Contribution to patients’ hand functions	78%	22%	–	–	29%	71%	–	–
	#27	#5	–	–	#6	#16	–	–
Contribution to patients’ cognitive functions	33%	22%	–	45%	57%	14%	–	29%
	#13	#2	–	–	#6	#2	–	–
Trust in the rehabilitation system social skills	44%	44%	12%	–	57%	29%	–	14%
	#22	#47	#2	–	#5	#5	–	–
Comparison to human therapist	33%	45%	22%	–	57%	14%	14%	14%
	#23	#37	#14	–	#7	#7	#5	–
Previous experience with technology	78%	11%	–	11%	71%	29%	–	–
	#18	#7	–	–	#16	#2	–	–
Tolerance to failures	56%	44%	–	–	57%	28%	–	14%
	#7	#9	–	–	#10	#7	–	–
Patient propensity to trust	33%	11%	–	56%	14%	28%	–	57%
	#3	#1	–	–	#1	#3	–	–
SAR’s perceived safety	11%	–	–	89%	–	–	–	100%
	#1	–	–	–	–	–	–	–
Patient optimism/pessimism	56%	22%	–	22%	71%	14%	–	14%
	#20	#14	–	–	#17	#2	–	–
Total per opinion	53%	43%	4%	–	49%	40%	11%	–
	#227	#186	#16	–	#94	#76	#22	–

In front of each category at the top is the percentage of patients according to their type of statement in relation to the category. At the bottom of each cell is the total number of statements encoded for the category. The cells in the columns titled “n/a” correspond to participants who did not take a stance (supporting/opposing/ambivalent) on the topic

Finally, the textual information was translated into quantitative terms [37] by calculating the frequency of statements for each category and sub-category, and the distribution of patient attitudes in each category, presenting a comparative analysis between and within the groups. This method enables to turn the unstructured data found in the interviews into a detailed description about important aspects of the situation under consideration [15].

## Results

**Fig. 4:**
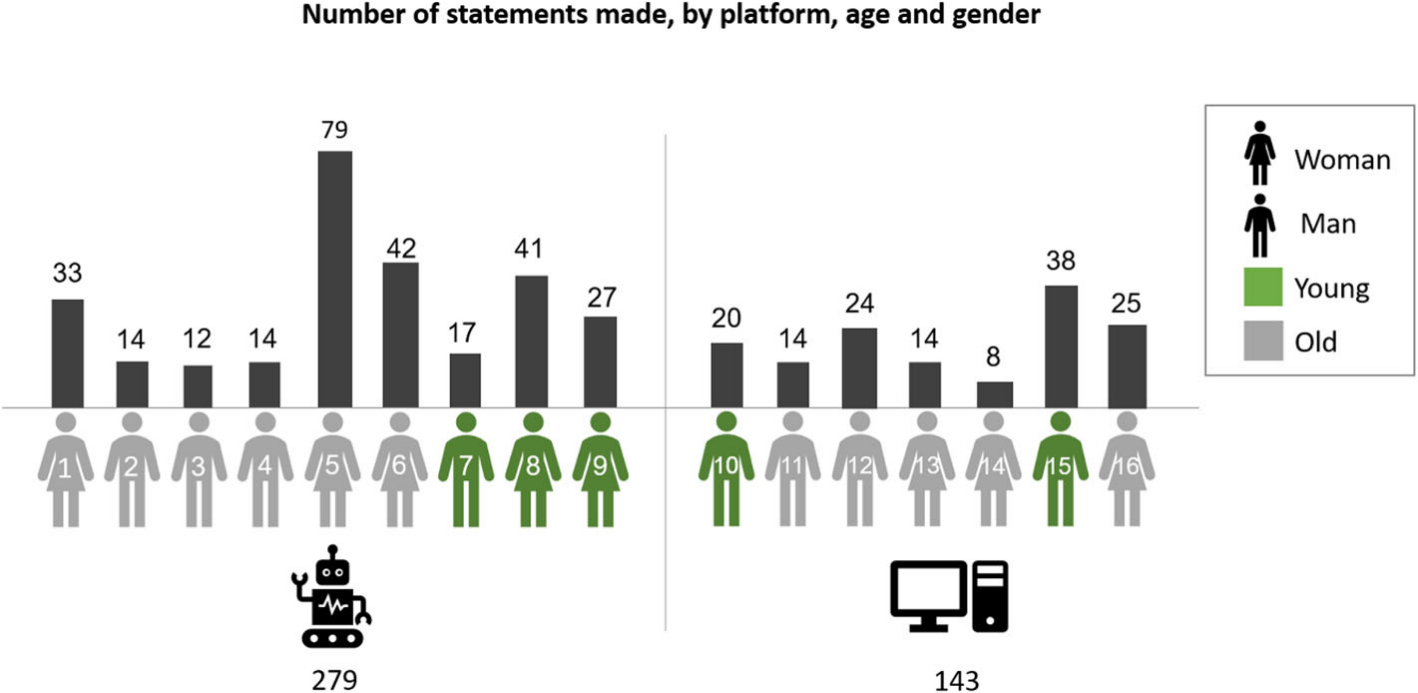
Number of statements made, by platform, age and gender. A total of 297 statements were made by nine participants in the SAR group (left), and 143 statements were made by seven participants in the Computer group (right). Shown here are the number of statements, as well as the number of interviews, per participant. Participants over the age of 60 were considered to be in the “older” group; participants under the age of 60 were considered to be in the “younger” group

Analysis of 16 patients’ interviews yielded 423 relevant statements, of which 279 were expressed by patients who practiced the rehabilitation platform operated by SAR (“SAR group”) and 143 by computer patients (“Computer group”). On average, SAR patients expressed their opinion more extensively, with the number of statements made by members of each group spanning a wide range (SAR group: 12–79, mean ± SD 66 ± 10; Computer group: 8–38, mean 20 ± 9) (see Fig. 4).

In addition, the type of interview carried out (semi-structured), and the emphasis given to the free expression of the interviewees, yielded a different number of statements on the various topics. As a result, a different number of statements were obtained in each category. Categories based on factors previously identified in the literature as influencing the level of trust in robots (a priori coding) yielded 280 statements (44%), and categories based on “open coding” yielded 341 statements (56%). (See Table 1 for a distribution of the number of statements according to categories). What follows is a description of the findings by main categories. All quotes from the interviewees were translated from Hebrew with an emphasis on maintaining the authenticity of their original statements.

### Patients Satisfaction with the Rehabilitation Activity

Most SAR-group patients (78%) expressed satisfaction with the rehabilitation intervention (i.e., the therapeutic procedure), as exemplified by the following quotations:

I realized that by getting the [therapy with the] robot, I won a prize (*Patient 06*)

With the robot it was interesting and entirely different from the other [rehabilitation] treatments I have had so far (*Patient 08*)

It is noteworthy that patients who expressed dissatisfaction with the treatment with the SAR (22%) were blunter in expressing their dissatisfaction, as revealed in a total of 38 opposing expressions by two unsatisfied patients (19 expressions on average per each), compared to a total of 45 supportive expressions by seven satisfied users (6.4 statements on average per each). The following quotation exemplifies an opposing expression:

The robot did not contribute anything to my rehabilitation (*Patient 09*)

Unlike patients in the SAR group, none of which were abivalent about the robot-assisted intervention, Computer-group patients expressed a less decisive position which was divided between satisfied (43%), dissatisfied (14%) and ambivalent (43%).

## Trust in the Rehabilitation System’s Functionality

Most SAR-group patients (78%) expressed satisfaction with the rehabilitation system’s functionality; i.e., its usefulness and capability to perform its intended goal of guiding the rehabilitation task, as exemplified by the following quotations:

With the robot I did things [exercises] better than in [conventional] occupational therapy (*Patient 02*)

I think the robot is very helpful because I really do with it [exercises], I would not do on my own (*Patient 08*)

A one-to-one match was found between SAR-group patients who expressed satisfaction with the rehabilitative activity in general, and with the SAR’s usefulness and capability to perform its rehabilitation task. Similarly, a one-to-one match was found between the SAR-group patients who expressed dissatisfaction with the rehabilitative activity and a lack of confidence in the SAR's usefulness. Unlike patients who exercised with the SAR, Computer-group patients expressed divided opinions, with only 29% advocates, whereas the challengers stood out more, when compared to the SAR group (29% opponents, 29% ambivalent, 13% no opinion). They also expressed more blatant statements, as exemplified by the following quotations:

I do not feel there is anything medical that can help me in this treatment (*Patient 15*)

Eventually, [conventional] physical therapy and occupational therapy were more effective (*Patient 16*)

### Contribution to Patients’ Motor and Cognitive Functions

The system enables the patients to practice both motor functions (movement of the impaired hand) and cognitive functions (memory and spatial perception).

#### Motor Function

Most patients who practiced with the SAR (78%) appreciated the contribution of the system to the rehabilitation of their impaired hand, and only 22% did not, as exemplified by the following quotations:

Supporters—“The device activates my hand; it is a smart system that contributes to the healing of my hand” (*Patient 07*)

Opponents—“I did not feel it healed my hand” (*Patient 05*)

Yet only a minority of patients who practiced with the computer (29%) expressed a supportive opinion. The opponents among Computer-group patients (71%) stood out more with more dissenting statements like:

It did not improve my hand at all (*Patient 12*).

Supportive statements by Computer-group patients are exemplified by the following quote:

The computer gave me this option to activate the hand (*Patient 10*)

#### Cognitive Function

A minority of patients who practiced with the SAR (33%) expressed a proponent opinion toward the system’s contribution to the improvement of their memory (22% opposing, 45% no opinion). Unlike the majority of patients who practiced with the computer (57%) who expressed a supporting opinion regarding this issue (14% opposing, 29% no opinion).

It should be noted that in general the number of statements about the contribution to memory improvement was relatively low, only 23 statements, compared to 54 statements about the contribution to the motor function of the hand.

### Trust in the Rehabilitation System’s Social Skills

Opinions about SARs’ social skills (i.e., its human-like behavior and ability to produce recognizable intentions, as well as other social behaviors, such as humor) among patients were not conclusive and were divided equally between supporters (44%) and opponents (44%) and a minority (12%) who were ambivalent. The quite low grade that was given to the SAR’s social skills is salient in comparison to the relatively high proportion of SAR-group patients (78%) who expressed a proponent assessment of the SAR’s functional skills. Following are illustrative expressions for proponent and opposed reference to the robot's social skills:

Supporters—“It [the SAR] is like a nice person” (*Patient 02*); “The robot was very kind, patient and nice” (*Patient 27*)

Opponents—“It is not as pleasant as with a human being” (*Patient 05*); “Machines cannot generate emotional relations” (*Patient 07*)

It is worth noting that patients who expressed an opposed attitude towards the SAR’s social skills were more outspoken in expressing their opposing opinion as expressed by a total of 47 opposing expressions compared to a total of 22 proponent ones. However, compared to SAR-group patients, those who exercised with the Computer expressed a different viewpoint. Most of them (57%) expressed a proponent opinion about the Computer’s social skills (29% opposing, 14% no opinion), although the total number of coded statements on the subject by Computer-group patients (# 10) was relatively low compared to the number of statements on this subject (# 71) by patients who exercised with the SAR.

### Comparison to a Human Therapist

While the rehabilitation platform is meant to augment treatments by a therapist and not replace them, a comparison between the sessions with the platform and sessions in the standard care with a human therapist emerged in the interviews. A significant percentage of the SAR-group patients (45%) expressed a preference for a human therapist, while a third (33%) expressed a preference for a SAR therapist, and 22% had no preference between the two. The following are illustrative expressions of preference for a human therapist versus a preference for treatment using a SAR:

Human therapist preference—“I would like a human being sitting in front of me and not a robot” (*Patient 06*); “A robot will never replace a human being” (*Patient 07*)

SAR therapist preference—“This robot is more interesting than a human being” (*Patient 02*);

No preference—“There is no difference between a human [therapist] and a robot” (*Patient 03*); “The robot and the human [therapist] are the same” (*Patient 04*)

In contrast, the position of Computer-group patients was slightly more decisive and most of them (57%) supported computer therapy (14% opposing, 14% ambivalent, 14% no opinion) as illustrated by the following quotations:

The computer is more efficient; it can assist therapists and save time (*Patient 16*)

For this matter as well, the total number of coded statements on the subject by Computer-group patients (#19) was relatively low compared to the number of statements on this subject (# 74) of patients who exercised with SAR.

### Other Factors Affecting Trust

Analysis of patient statements revealed several factors which are commonly perceived in the HRI literature as affecting trust in robots, including: (1) users’ previous experience with technology, (2) tolerance to robot failures, (3) users’ propensity to trust (i.e., tendency to trust people), and (4) their perception of the robot’s safety, that is, it will not harm them [3].

#### Previous Experience with Technology

Most SAR-group patients (78%) and most Computer-group patients (71%) reported positive attitudes towards new technologies, or prior experience with computers and smartphones. Except for one case (out of 16), no relationship was found between a proponent approach to new technologies and satisfaction or dissatisfaction with the rehabilitative activity or trust or distrust of the system.

#### Tolerance to Robot Failures

The statements of SAR-group patients regarding system failures (i.e., errors, breakdowns, disruption etc.) were divided roughly similarly between those who did not attribute to it significant importance or tended to contain them (56%), and those who considered it as an issue (44%). Similar was the distribution of opinions among Computer-group patients. The total number of statements coded on this subject (# 33) was relatively low. The following are illustrative expressions of patients who tend to contain system failures and of those for whom system failures are an issue:

Patients who tend to contain system failures—“Humans also have bugs” (*Patient 07*); “Despite the errors, I still think one can accept his opinion and trust him” (*Patient 08*)

Patients who consider system failures as an issue—“He is mistaken too many times” (*Patient 05*); “There were failures in almost every session” (*Patient 09*)

#### Patients Propensity to Trust and SAR’s Perceived Safety

There were very few testimonies on these issues. Regarding the patients’ propensity to trust people in general. Only eight statements were found, several indicating a tendency to a priori trust and others a general suspicious attitude (these statements were uttered as self-descriptions, and not regarding these participants’ trust/suspicion towards technology). With regard to SAR’s perceived safety (i.e., patients’ concerns regarding the safety of the robot) only one statements was identified asserting a sense of safety with the SAR, was expressed by the statement: “I am willing to be left alone with him” (*Patient 08*).

### Age and Gender

#### Gender

**Fig. 5:**
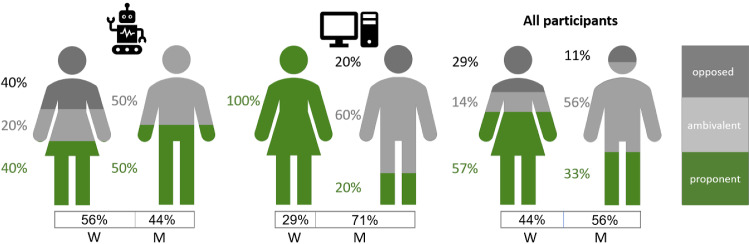
Stance by gender. The statements made by participants were coded into “proponent” (the percent of supportive statements within the group, shown here in green), “ambivalent” (the percent of ambivalent statements within the group, shown here in gray), and “opposed” (the percent of negative statements within the group, shown here in black). The three categories are shown per gender group (*W* women; *M* men) within the SAR group (left), the Computer group (middle), and across all participants (right). The percent of the participants from each gender in each group is shown on the bottom

The interviewed group included 16 patients, of whom 9 (56%) were men and 7 women (44%). Overall, women were more supportive towards the system (57% supportive, 14% ambivalent, 29% opposed), while men were more ambivalent (33% supportive, 56% ambivalent, 11% opposed). However, opinion distribution by gender differed between the SAR and the Computer groups: Men practicing with the SAR were more proponent (50% proponent, 50% ambivalent), while women’s position was more varied (40% proponent, 20% ambivalent, 40% opposed). In contrast, Computer-group woman patients had a proponent attitude (100%), while men were more ambivalent (20% proponent, 60% ambivalent, 20% opposed). A summary of the distribution of patients’ attitudes by Gender is shown in Fig. 5.

#### Age

The ages of the interviewed patients ranged between 30 and 74 years, mean 58 ± 13 years; SAR-group patients age range was 30–68 years, mean 52 ± 11 years; Computer-group patients age rang was 42–74 years, mean 66 ± 10. We divided each group into two age sub-groups: “older” patients and (relatively) “younger” patients; The allocation into age groups was as customary by the World Health Organization [43], thus patients below 60 were assigned to the “younger” group and the rest to the “older” group. The results of the analysis indicate that overall, the older patients were more proponent (56% proponent, 33% ambivalent, 11% opposed) than the younger patients (29% proponent, 43% ambivalent, 29% opposed). Similar results were also observed within the groups (both SAR and Computer). A summary of the distribution of patients’ attitudes by Age is shown in Table 3.

**Table 3:** Stance by age

	Age group		Proponent	Ambivalent	Opposed
All patients	Older	56%	56%	33%	11%
	Younger	44%	29%	43%	29%
SAR patients	Older	33%	67%	–	33%
	Younger	67%	33%	50%	17%
Computer patients	Older	86%	50%	50%	–
	Younger	14%	–	–	100%

### Summary of Patients Tendency to Trust the System

The interviewed group included 16 patients, of whom 56% practiced with a SAR and 44% with a computer. Most patients expressed satisfaction with the rehabilitation activity (62% proponent, 19% opposed, 19% ambivalent), especially among the patients practicing with the SAR (78% proponent, 22% opposed) while patients practicing with the Computer were less convinced (43% proponent, 14% opposed, 43% ambivalent). In particular, patients who exercised with the SAR appreciated the system’s contribution to the improvement of their arm’s motor abilities (78% proponent), whereas patients who exercised with the Computer did not as much (71% opposed), and preferred its contribution to the improvement of their cognitive (memory) abilities (57% proponent). In contrary, patients who exercised with the SAR were less impressed by the cognitive rehabilitation aspect of the system (only 33% proponent). In general, a greater trust was given to the system’s functionality (56% proponent, 25% opposing, 12% ambivalent, 6% no opinion) compared to its social capabilities (50% proponent, 38% opposing, 6% ambivalent, 6% no opinion). However, there was a significant difference between the groups. While the SAR group was more impressed with the system’s functional skills (78% proponent for functional skills, 44% proponent for social skills), the Computer group less appreciated this aspect, but acknowledged its social skills (29% proponent for functional skills, 57% proponent for social skills). Most patients tended to contain the system failures (56% tolerant, 38% intolerant, 6% no opinion), with roughly similar results between the groups: SAR (56% tolerant, 44% intolerant) and the Computer group (57% tolerant, 28% intolerant, 14% no opinion). Analysis of attitudes that compares views on rehabilitation treatment with the new technological tool (SAR or Computer) to treatment by a human therapist revealed that most SAR-group patients tended to prefer a human therapist (45% for human, 33% for SAR, 22% ambivalent), while Computer-group participants tended to prefer Computer therapy (57% for computer, 14% for human, 14% ambivalent, 14% no opinion). Relatively older patients were more supportive of the system than younger and women more than men. The analysis also revealed patients’ views on their rehabilitation prospect. Related statements were classified to “optimistic” attitude versus a “pessimistic” one, or expressions of “satisfaction” versus “frustration”. The categorization resulted in 53 statements, most of which revealed an optimistic attitude among SAR-group patients (56% optimistic, 22% pessimistic, 22% no opinion) and even more optimistic among Computer-group patients (71% optimistic, 14% pessimistic, 14% no opinion). Following are illustrative expressions for optimistic and pessimistic expressions:

Optimistic—“I am in a better state than I used to be” (*Patient 13*); “it takes time, but things are improving” (*Patient 08*)

Pessimistic—“My life is over” (*Patient 05*); “There is no improvement in my physical condition” (*Patient 12*)

**Fig. 6:**
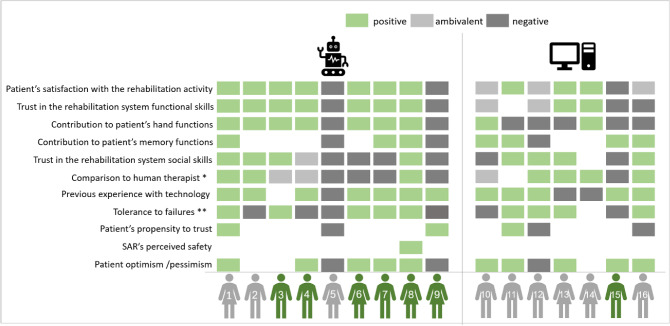
A summary of the distribution of patients’ opinions, by main categories. Patients’ statements with respect to each of the topics listed on the left were categorized as either positive (green), ambivalent (light gray), or negative (dark gray). If the individual did not make any statements relating to the topic, it is left blank. *Comparison to human therapist: SAR or computer preference is denoted by the green color; human therapist preference is denoted by the dark gray color; no preference is denoted by the light gray color; **Tolerance to failures: accepting of system failures is denoted by the green color; not accepting system failures is denoted by the dark gray color. (Color figure online)

The findings listed in Fig. 6 indicate that a relatively high compatibility exists between SAR-group patients, who expressed a general optimistic attitude, and their satisfaction with the rehabilitative activity with the SAR. And a one-to-one match was found between SAR-group patients, who expressed a general pessimistic attitude, and their dissatisfaction with the rehabilitative activity with the SAR. No such matching was found among Computer-group. A summary of the distribution of patients’ opinions, by main categories, is shown in Fig. 6.

## Discussion

We investigated post-stroke patients’ attitudes to a rehabilitative system, which provides instructions and feedback by a SAR or by a computer interface [13]. It aimed to find out users’ perspectives on factors related to HRI trust when the patient is in a direct interaction with a SAR. To that end, we conducted 29 interviews with 16 post-stroke patients who participated in a rehabilitation process in which the SAR or computer systems were involved. Research involving people with disabilities are characterized by a relatively small sample [38]. It is generally acceptable to recruit 5–10 participants for a study involving people with disabilities [15]. The relatively large number of patients recruited for this study (16) enhances the validity of its findings.

The findings of this study advance our understanding of users’ attitudes toward advanced technology such as SARs and enhance the understanding of trust in SARs’ application for therapeutic rehabilitation. The results support the assertion that SAR could have an added value in the rehabilitative care of stroke patients.

### Patients’ Satisfaction with the Rehabilitation Activity

Most SAR-group patients expressed a positive attitude toward the system. Two out of the nine patients in this group expressed a wide-ranging positive attitude in all categories, while the rest of the supporters expressed a moderately positive general opinion, varying among categories. A minority (22%) of the SAR-group patients expressed a general negative, and very blatant opinion, wide-ranging in all categories. The stance of the minority suggests that it is important for SAR developers to carefully examine and customize it to the profile of their potential users, taking into account their different sensitivities to the various trust factors. For example, to prioritize robots’ functionality over its sociality. It confirms the call for a higher level of personalization in designing SAR for rehabilitation [3]. The positive attitude of most SAR-group patients regarding the system was consistent with their satisfaction with the rehabilitation benefit they experienced. This result is compatible with previous findings [9, 20] that when users expressed greater satisfaction, their trust was higher. Although most Computer-group patients expressed a positive attitude toward the system, their position was more complex and heterogeneous compared to SAR-group patients, with greater variability among categories. This is reflected mainly in a lower degree of satisfaction with the rehabilitation activity (43% supporting) compared to SAR-group patients (78% supporting). As both groups experienced the same rehabilitation activity, and the only difference was the operating platform (SAR with its associated unique embodied features and related behavior, as opposed to a standard computer), it is conceivable to assume that the SAR had an added value to the rehabilitative activity compared to the Computer. This finding supports the claim that robots can offer advantages over a computer tablet in healthcare [36]. However, the factors that give rise to the difference between the two groups should be further investigated to better understand the foundations for the SAR potential added value. As discussed below, the embedded social skills, associated with the SAR, did not explicitly explain the difference between the groups.

Satisfaction (or dissatisfaction) inductively emerged as a category while employing the open-coding procedure. Of note is that Hancock et al. [9] added in their recently updated meta-analysis on antecedents of trust in robots that “if users expressed greater satisfaction, trust also improved”. It is interesting to note that satisfaction does not play a central role in the Technology Acceptance Model (TAM), as it is regarded as difficult to measure [44]. Our results suggest that *satisfaction with the rehabilitation activity* should also be added to the trust model in the context of rehabilitation.

### Trust in the Rehabilitation System Functional Skills

The analysis of the factors affecting patient satisfaction with the system revealed that among the SAR-group patients, trust in the system functionality prevailed (78% supporters). This finding on the importance of the SAR’s usefulness—its functional ability to carry out its task (performance), is consistent with previous studies [9, 11, 23, 45, 46]. The relatively unequivocal attitude of SAR-group patients stands out in contrast to the high variability evidenced among Computer-group patients’ attitudes toward the functionality of the system, of which only a minority (29%) expressed a supportive approach. These differences in attitudes support the view that SARs have an added value that may play a positive role in the rehabilitative care of stroke patients [3] as they may be acceptable to patients as caregivers more than mere standard computers.

### Contribution to Patients’ Motor and Cognitive Functions

Patients’ testimonies indicate that relatively more SAR-group patients reported experiencing a significant contribution to their impaired hand rehabilitation (78% supporters) compared to a lesser contribution perceived to their memory rehabilitation (33% supporters) (see Fig. 6; Note that some of the patients did not make statements on these topics). The lower perceived contribution to memory rehabilitation can be interpreted as lower significance given by patients to this element of the system, or that their perception of the successful contribution to their hand function dominated their awareness of the systems’ dual goals, or less success of the roboticists in realizing this objective. Evidence from Computer-group patients may suggest a probable explanation of the differences in perception of the system’s contribution to the motoric element relative to the cognitive one. In contrast to SAR-group patients, relatively more Computer-group patients reported experiencing a significant contribution to their memory rehabilitation (57%) and less in relation to their hand rehabilitation (29%). Of note is that the intervention was not designed specifically to train memory skills, but rather to support the motor rehabilitation of their upper arm. It is thus interesting to note the perceived benefit to their memory skills that the patients reported. Importantly, the platform itself, and the sets of exercises, as well as the instructions and feedback were *identical* for the two groups. The only difference was which device provided the instructions and feedback—a SAR with its unique embodied features and related behavior—or a Computer. However, the participants in the two groups report vastly differing perceptions of their experiences. A plausible explanation is that the SAR’s physical gestures and facial expressions induce a higher sense of physical activity, compared to the interaction with a computer which gives a lesser sense of physical activity and induce a higher sense of cognitive activity. These differences need to be further investigated in order to understand which rehabilitative goals are pertinent for treatments using a SAR.

### Trust in the Rehabilitation System Social Skills

Contrary to the trust that most SAR-group patients expressed regarding the system’s functionality (78% supportive), they expressed less appreciation of its social skills (44% supportive). The attitude regarding the SAR's social skills can be interpreted as a lower importance given by the patients to this element of the system. These results are compatible with previous research according to which older adults have expressed preference to robot’s technical functionality, as the most important characteristic of SARs used in aged-care settings, over its appearance and social behavior [47]. Perhaps surprisingly, the Computer-group patients presented a different, and a more positive stance regarding the system’s social skills (57% supporters). This finding is consistent with the “media equation theory” [48], according to which people treat media such as television or computers as if they were humans despite being aware of their artificiality. Although they had little to say on the subject (only ten statements), their different view from SAR-group patients may imply that the latter had high expectations to experience more significant social abilities of the SAR which were not realized. Computer-group patients, on the other hand, presumably had a low level of expectations in this aspect, but the verbal and visual feedback that the computer presented, as part of the rehabilitative activity, induced a sense of social abilities. The relatively low appreciation to the SAR’s social skills challenges the prevalent claim in the literature that robot users will better trust it as a partner if it succeeds in producing familiar human-like properties such as gestures, facial expressions and other accepted behavioral and social cues that set up social relations [9, 49]. The same was asserted with regard to SAR for rehabilitative activities [3]. In contrast, our findings in this regard resonate the claim that social robots should not be automatically designed anthropomorphically as this may increase the chance of disappointment if the robot does not function socially as expected by users [50]. Our findings are also consistent with the study by Gaudiello et al. [51] that showed that users trust robots more with tasks that require decisions on functional issues and less with tasks that require decision on social issues and vice versa, the minority of users who trust robots on social issues show significant distrust in robots on functional issues. Patients’ reservations about the SAR’s social skills call into question roboticists’ efforts to anthropomorphize rehabilitative SARs, and suggest that in designing SAR, they should carefully consider users’ priorities and whether, and to what extent, SAR’s social skills are applicable to their needs.

### Comparison to a Human Therapist

While the explicit goal of the platform tested here is to augment treatments by a therapist, and not replace them, a comparison between the sessions with the platform and sessions with human therapist did emerge in the interviews. In line with the finding that SAR-group patients expressed less trust in the SAR’s social skills, the majority of them (45%) also expressed a tendency to prefer a human therapist over a SAR. Though others in the group (33%) were supportive of the SAR implementation (22% were ambivalent). In contrast, most Computer-group patients (57%) gave preference to a system operated by a computer over a human therapist. The preference of Computer-group patient in this matter is unclear and should be further researched. With respect to the indecisive stance of SAR-group patients, it seems that there are stroke patients who are willing to integrate rehabilitation exercises by a SAR, but apparently further developmental effort is required to adjust the rehabilitative SAR to the requirements of a wider patient population. Further, although more SAR patients expressed a preference to a human therapist, it is important to emphasize the stance of those in doubt and the minority that preferred to be treated by a SAR. It could support a claim that SARs can be integrated into the process of rehabilitation to augment the current range of therapeutic options [3]. It encourages the approach that calls for collaboration in rehabilitative care of a human therapist and SAR to maximize the relative benefit of each. Thus, SAR can be used to interact with patients in the absence of a therapist to encourage practice between sessions [4], and could be more appropriate for certain rehabilitative tasks, such as performing repetitive movements. Meanwhile, the human therapist may provide the patient with the depth of interpersonal interaction, comfort, compassion and empathy [3, 12]. However, based on the findings of this study, it appears that the use of a SAR, as an augmented rehabilitative approach, is not pertinent for every patient.

### Previous Experience with Technology

The majority of SAR-group participants (78%), as well as Computer-group participants (71%) have had previous experience with technology devices (e.g., computers, smartphones), and had a positive view about them, but no compatibility was found between this general attitude and their stance toward the system or its technological operator. It is consistent with findings about activities with other types of robots according to which early experience with technology was not found to be an important condition for trust in robots [9]. However, it does not confirm what was advised with respect to rehabilitation patients [3] that in order to trust a therapeutic SAR it is better that they have previous experience with advanced technology.

### Other Factors Affecting Trust

Other factors, considered in the literature as mediators of trust in SAR such as level of system failures [3, 34, 35], safety concerns [3, 31] and individual propensity to trust [3, 33] were seldom identified in patients’ statements, but their total number was limited, thus not conclusive and should be further investigated.

The majority of SAR-group participants who expressed a positive attitude toward the system tended to be more optimistic about their recovery process, while patients with negative attitude also expressed a pessimistic view about their recovery state. No such compatibility was found among Computer-group participants. Nevertheless, and particularly with respect to SAR, the findings suggest the importance of characterizing potential users for treatment with SAR rehabilitation. This claim is also supported by participants’ related demographics analysis. The opinion of the participants in the relatively older-age group was more positive while the younger-age were more critical. It supports previous cases showing that older adults are not necessarily less receptive to robots as younger users [52–54]. We also found gender to play a role in users’ preferences: Women practicing with the SAR were more critical than men. This finding is contrary to previous research [55] which demonstrated that women reported higher trust and perceived trustworthiness of the robot relative to men.

Findings on the effect of demographic aspects and user personality traits indicate the significance of characterizing potential users for SAR therapy and is compatible with contemporary research which identified user’s characteristics and personality as having a significant impact on human trust in robots [9, 20]. Our findings support this claim and emphasize its importance in the context of SAR engagement in rehabilitation treatments.

In this research we conducted and analyzed 29 interviews with 16 post-stroke individuals who trained with either SAR or Computer rehabilitation system in order to characterize their HRI or HCI experience and examine factors that affect their trust in the system. The participants in the two groups expressed different preferences in relation to the various aspects of the system: patients practicing with the SAR expressed satisfaction with the rehabilitation activity, while patients practicing with the Computer were less convinced of its usefulness. The former preferred the system’s contribution to the improvement of their arm’s motor abilities, whereas the latter preferred its contribution to the improvement of their cognitive (memory) abilities. Similarly, there were varying preferences within the groups, as detailed above. Overall, it appears that SAR-group patients, as a group, were more willing to accept the system. This result supports the notion that SARs can augment rehabilitative therapies beyond a standard computer [3, 56].

The trust factors revealed in our study partly overlap with previously identified trust factors, with refined factors identified in the specific case of post-stroke patients. As for the factor *system’s functionality*, our findings verify the importance of the robot’s functional ability to carry out its task in the context of post-stroke rehabilitation, and are consistent with previous studies regarding this trust factor [9, 11, 23, 45, 46]. Our findings, however, challenge the prevalent claim in the literature that the SAR’s *social skills* (human-like properties such as gestures, facial expressions, and other behavioral and social cues) will increase trust [3, 9, 49, 50]. As for the factor *user’s prior experience with technology*, we found no correspondence between post-stroke patients’ prior experience with technology and their stance toward the system. This finding is consistent with previous work in other robotic contexts, as in the case of a human operating a robot, which also found that early experience with technology was not an important condition for trust in robots [9]. As such, it does not confirm the suggestion that patients will be more likely to trust a therapeutic SAR if they have previous experience with advanced technology [3]. We did not find the factors *tolerance to system failures*, *user’s safety concerns*, and *user’s propensity to trust* as central in trusting the robot in the context of post-stroke rehabilitation; These findings contrast with literature which defines these as mediators of trust in robots [3, 31, 33–35]; Our findings verify that a positive attitude regarding the system was consistent with patients’ satisfaction with the *rehabilitation benefit they experienced*. This result is compatible with previous findings [9, 20] showing that when users expressed greater satisfaction from the system, their trust was higher; We thus suggest that *experiencing rehabilitation benefit* should be added as a factor affecting trust in HRI in the context of rehabilitation. We further present the distinctions between patients’ perceived benefits of a robot vs. a standard computer as non-human operators in the specific context of rehabilitation. Our findings reveal that it is conceivable to assume that robots have an added value to the rehabilitative activity compared to the standard computers, consistent with the claim that robots can offer advantages over a computer tablet in healthcare [36]. Our findings present a more nuanced difference between a robot and a standard computer for post-stroke rehabilitation, which previous work did not discuss. For instance, patients’ sensitivity to the specific task-operator pairings: preferring motor-skill rehabilitation by a robot while favoring cognitive rehabilitation with a standard computer.

## Conclusions

As non-human operators have been offered as a tool to assist post stroke individuals to perform their exercise during their rehabilitation process, it is essential that these non-human operators will be trusted by patients. By using a qualitative research method—extended interviews with stroke patients, who underwent a long-term rehabilitation with a non-human operator in their conventional therapeutic setting—our study reveals users’ perspectives regarding factors affecting trust in SAR and computer interface in their rehabilitation context.

The results support the assertion that a SAR has an added value in the rehabilitative care of stroke patients. We found an effect of demographic aspects (e.g., age, gender) and user personality traits, such as their general attitude (e.g., optimistic/pessimistic) about their overall recovery process. Yet, we did not find other factors specified in the literature with respect to robot-related (failure rate and its human-like behavior and anthropomorphism) and human-related (prior experience, personality traits, propensity to trust) as central factors in generating trust and encouraging compliance with the SAR.

Although our findings support the opinion that SARs can augment rehabilitative therapies beyond a standard computer, a detailed analysis reveals how related factors selectively affect users’ trust in each platform; In this context, the prominent finding is that patients in the SAR group appreciated the system’s contribution to the improvement of their arm’s *motor* abilities whereas patients in the Computer group preferred its contribution to the improvement of their *cognitive* abilities.

Patients’ attitudes, as evidenced by interviews, can serve as guidelines for further development of non-human operators. A prominent guideline that came out of the current work is users’ preference of the SAR’s *functional performance* over its anthropomorphized social skills.

Though most participants stated a supporting opinion about the system, the opinions of the outspoken opponent minority are well detailed in this paper. It is important to present their views in a reflective manner, in order to help researchers and clinicians to make a decision about who is suitable for training with a system and who is not, and what adjustments should be made to improve the system and to promote personalized neurorehabilitation [57] to patients’ need.

The main contributions of this work are: (1) revealing the patients’ perspective on long-term training with a non-human operator; (2) distinguishing between the perceived benefits of a SAR vs. a Computer as non-human operators in the context of rehabilitation; (3) identifying factors that affect users’ acceptance of the system—most prominently, patients who perceived the system as beneficial to their motor/cognitive improvement, tended to accept the system; and (4) implementing a qualitative methodology, using multiple, in-depth interviews during and after the long-term in-the-wild intervention program.

## Limitations and Future Research

In our study, participants were interviewed at different time points, a different number of times each (Fig. 2). The overall positivity or negativity with regards to using the technological platform (Fig. 6) may be impacted by both the individual’s personal propensity to have a positive or negative outlook as well as by the time that passed since the intervention was held. This might affect how people recall the interaction: the positivity in memory bias may cause people to remember the intervention as more positive than they felt at the time of the intervention. While roughly half of the interviews were held within the 5–7-week period of the intervention (16 out of 29 interviews), and thus less subject to potential effects of a memory bias, it would be instructive to conduct future experiments with this in mind, attempting to hold similar numbers of sessions at specific time points, such that the effect of time can be estimated.

The trust of a patient in a robot in the context of rehabilitation is likely affected by the trust of their therapist in the robot, as well as the trust of their caregivers (e.g., a close family member) in it. These triadic, or potentially even quadratic relationships: SAR-patient-therapist(s)-caregiver require a further, separate investigation. Preliminary research on the attitudes of clinicians [2] and of family members [58] should be further elaborated upon.

As our focus in this work is on factors affecting trust in the use of technology for rehabilitation, we did not interview participants in the “control” condition of the RCT study, as they did not interact with the technological platform. We do, however, acknowledge the potential benefit of conducting interviews with control participants in future studies.

Interviews are an important source of insights and useful data that would otherwise be hard to capture. However, qualitative analysis has a high subjective component. Complementary studies, using various methodologies, will enhance our knowledge of patients' perspectives and their needs in a diverse rehabilitative context. Further extended interviews with stroke patients, in their conventional therapeutic setting over a long-term rehabilitation, are required to expand knowledge on how trust in non-human operators in the therapeutic context evolves over time, and to promote understanding of contextual factors, like what and how sociocultural factors play a role in the way people trust SARs in rehabilitation.

## Supplementary Information

Below is the link to the electronic supplementary material.

Supplementary file1 (DOCX 15 kb)

## Data Availability

Data will be made available on reasonable request.
