# Supplementary material for: Extended Interviews with Stroke Patients Over a Long-Term Rehabilitation Using Human–Robot or Human–Computer Interactions
Source: Int J Soc Robot. 2022 Sep 16;14(8):1893–911. doi: 10.1007/s12369-022-00909-7 (PMC9483483; doi:10.1007/s12369-022-00909-7)
Supplement: Supplementary file 1 — Supplementary file1 (DOCX 15 kb) [file 12369_2022_909_MOESM1_ESM.docx]

Supplementary Materials

**Extended Interviews with Stroke Patients Over a Long-term Rehabilitation Using Human-Robot or Human-Computer Interactions**

Yaacov Koren, Ronit Feingold Polak and Shelly Levy-Tzedek*

The inclusion criteria of the RCT study – and hence for the current study – were as follows: (1) First unilateral stroke, confirmed by imaging; (2) age: 18-85; (3) Mini Mental State Examination (MMSE) score ≥24/30 (for participants ≥ 65yrs) [1] or the equivalent Montreal Cognitive Assessment (MoCA) score ≥ 23/30 (for participants < 65 yrs) [2]; (4) Fugl-Meyer Upper Extremity Assessment (FMA) [3, 4] score ≥16/60 (higher score indicates less impairment; a score ≤16/60 indicates the patient does not have the capacity to reach and grasp objects); (4) no excessive pain in the upper limb, defined as ≤ 4 on a scale of 10. Exclusion criteria were as follows: (a) Other neurological or musculoskeletal conditions affecting upper-limb movement (e.g., Parkinson's disease, unilateral neglect); (b) severe vision or sensory deficits affecting upper limb movements; and (c) aphasia impeding comprehension of simple instructions [5].

**References:**

1. Folstein MF, Folstein SE, McHugh PR: **“Mini-mental state”: a practical method for grading the cognitive state of patients for the clinician**. *Journal of psychiatric research* 1975, **12**(3):189-198.

2. Carson N, Leach L, Murphy KJ: **A re‐examination of Montreal Cognitive Assessment (MoCA) cutoff scores**. *International journal of geriatric psychiatry* 2018, **33**(2):379-388.

3. Fugl-Meyer AR, Jääskö L, Leyman I, Olsson S, Steglind S: **A method for evaluation of physical performance**. *Scand J Rehabil Med* 1975, **7**:13-31.

4. Woytowicz EJ, Rietschel JC, Goodman RN, Conroy SS, Sorkin JD, Whitall J, Waller SM: **Determining levels of upper extremity movement impairment by applying a cluster analysis to the Fugl-Meyer assessment of the upper extremity in chronic stroke**. *Archives of physical medicine and rehabilitation* 2017, **98**(3):456-462.

5. Levin MF, Liebermann DG, Parmet Y, Berman S: **Compensatory versus noncompensatory shoulder movements used for reaching in stroke**. *Neurorehabilitation and neural repair* 2016, **30**(7):635-646.
